# Hippocampal Expression of Cytochrome P450 1B1 in Penetrating Traumatic Brain Injury

**DOI:** 10.3390/ijms23020722

**Published:** 2022-01-10

**Authors:** Erik Lidin, Mattias K. Sköld, Maria Angéria, Johan Davidsson, Mårten Risling

**Affiliations:** 1 Experimental Traumatology Unit, Department of Neuroscience, Karolinska Institute, 171 77 Stockholm, Sweden; mattias.skold@ki.se (M.K.S.); maria.angeria@ki.se (M.A.); Marten.Risling@ki.se (M.R.); 2 Section of Neurosurgery, Department of Neuroscience, Uppsala University, 751 85 Uppsala, Sweden; 3 Department of Mechanics and Maritime Sciences, Chalmers University of Technology, 412 96 Gothenburg, Sweden; johan.davidsson@chalmers.se

**Keywords:** traumatic brain injury, sex differences, cytochrome P450 1B1, hippocampus

## Abstract

Hippocampal dysfunction contributes to multiple traumatic brain injury sequala. Female rodents’ outcome is superior to male which has been ascribed the neuroprotective sex hormones 17β-estradiol and progesterone. Cytochrome P450 1B1 (CYP1B1) is an oxidative enzyme influencing the neuroinflammatory response by creating inflammatory mediators and metabolizing neuroprotective 17β-estradiol and progesterone. In this study, we aimed to describe hippocampal CYP1B1 mRNA expression, protein presence of CYP1B1 and its key redox partner Cytochrome P450 reductase (CPR) in both sexes, as well as the effect of penetrating traumatic brain injury (pTBI). A total 64 adult Sprague Dawley rats divided by sex received pTBI or sham-surgery and were assigned survival times of 1-, 3-, 5- or 7 days. CYP1B1 mRNA was quantified using in-situ hybridization and immunohistochemistry performed to verify protein colocalization. CYP1B1 mRNA expression was present in all subregions but greatest in CA2 irrespective of sex, survival time or intervention. At 3-, 5- and 7 days post-injury, expression in CA2 was reduced in male rats subjected to pTBI compared to sham-surgery. Females subjected to pTBI instead exhibited increased expression in all CA subregions 3 days post-injury, the only time point expression in CA2 was greater in females than in males. Immunohistochemical analysis confirmed neuronal CYP1B1 protein in all hippocampal subregions, while CPR was limited to CA1 and CA2. CYP1B1 mRNA is constitutively expressed in both sexes. In response to pTBI, females displayed a more urgent but brief regulatory response than males. This indicates there may be sex-dependent differences in CYP1B1 activity, possibly influencing inflammation and neuroprotection in pTBI.

## 1. Introduction

Secondary injuries of traumatic brain injury (TBI) are initiated by biochemical changes that occur directly after primary injury [1] and are essential to manage for reduction of injury sequala. However, despite recent progress in the comprehension of the complex cascade of molecular events characterizing the secondary injury, there are no therapeutic agents available [2].

In a previous publication we showed regulation of genes including the Cytochrome P450 family in hippocampus and cortex after penetrating traumatic brain injury (pTBI) in rats [3], further analysis of that material showed Cytochrome P450 1B1 (CYP1B1) to be distinctly upregulated. Data extracted from this gene array material regarding the Cytochrome P450 family are presented in the Appendix A (Table A1).

CYP1B1 is a fundamental enzyme in both humans and rodents, influencing metabolic and inflammatory pathways as well as metabolizing multiple xenobiotics [4,5]. CYP1B1 metabolizes arachidonic acid (AA) to hydroxyeicosatetraenoic acids (HETEs), a group of inflammatory mediators that generate reactive oxygen species and activate apoptotic pathways [6,7]. Furthermore, CYP1B1 is a key extrahepatic metabolizer of neuroprotective 17β-estradiol (E2) [8]. E2 is a potent neuroinflammatory mediator, administration of E2 in male and ovariectomized female mice exposed to TBI reduces infarction size, edema and hippocampal apoptosis and improves outcome [9,10,11]. CYP1B1 also metabolizes progesterone, that has multifactorial neuroprotective properties that effect excitotoxicity, cerebral edema, and inflammatory cytokines [4,12]. However, evidence of both positive and negative effects of altered CYP1B1 protein levels or its products exist, suggesting that CYP1B1 acts on both beneficial and deleterious pathways [4,5,13,14]. CYP1B1 activity directly correlates to its essential electron donor Cytochrome P450 reductase (CPR), and deletion of this key redox partner results in loss of practically all CYP1B1 function [15,16].

Experimental rodent models have highlighted the importance of sex when studying TBI, as female rodents have a lower mortality when exposed to TBI [10]. Additionally, female rodents also exhibit reduced lesion area, cerebral edema, and cell death [17,18,19]. However, indications of sex-dependent differences and a beneficial effect of female sex hormones in rodent TBI models do not align with epidemiological studies of TBI in humans, where results are ambiguous [20,21,22,23].

Hippocampal pathology is central in several TBI sequala such as sleep disorder, cognitive disability, and memory impairment. The hippocampus consists of distinct subregions with unique intrinsic and extrinsic connections [24]. Cellular subpopulations within each subregion exhibit differences regarding circuity, gene expression as well as both electrophysiological and functional properties [25,26].

We have developed a model utilizing a high probe velocity and shape to simulate a clinically plausible pTBI [27,28,29]. CYP1B1 is believed to impact multiple molecules part of the secondary injury of pTBI, by producing inflammatory mediators HETEs [6,7] and metabolizing neuroprotective E2 and progesterone [4,5]. No previous studies have studied hippocampal CYP1B1 mRNA expression in the context of TBI or sex. In this pilot study, we therefore aim to map the presence of hippocampal CYP1B1 in male and female rats. Furthermore, we aim to study the effect of pTBI on CYP1B1 mRNA expression and if indications of sex-dependent differences after injury exist.

## 2. Results

Due to the nature of this explorative pilot study, with multiple small groups aimed at providing information for future studies, groups are underpowered and statistical conclusions should be drawn with caution. Results indicating changes in expression following pTBI and sex-dependent differences are preliminary indications until confirmed by future studies with greater power.

### 2.1. In Situ Hybridization

**Figure 1:**
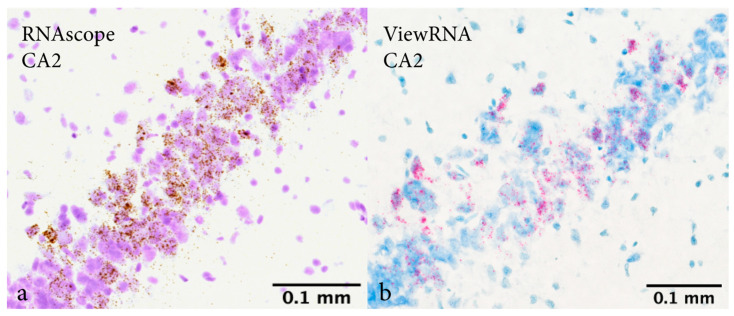
Representative images from ipsilateral subregion CA2 using RNAscope and ViewRNA. Images captured at 20× magnification from the same female rat subjected to penetrating traumatic brain injury and a survival time of 3 days. Captured at Bregma: −3.84 mm. Image (**a**) shows CYP1B1 mRNA marked in brown (RNAscope) and a cresyl violet counterstaining. Image (**b**) shows CYP1B1 mRNA marked in red (ViewRNA) and a methyl green counterstain. Expression was greatest in CA2 irrespective of sex, intervention and survival time using both RNAscope (**a**) and ViewRNA (**b**). Abbreviations: CA2: Cornu Ammonis 2.

In-situ hybridization detected CYP1B1 mRNA in all five hippocampal subregions. However, interregional differences were observed. Irrespective of sex, intervention, and survival time, CYP1B1 mRNA was highest in CA2 and lowest in DG. Quantification and analysis were performed on results from RNAscope and ViewRNA was used as a control to confirm our findings. The same pattern of expression was observed using both RNAscope and ViewRNA (Figure 1).

**Figure 2:**
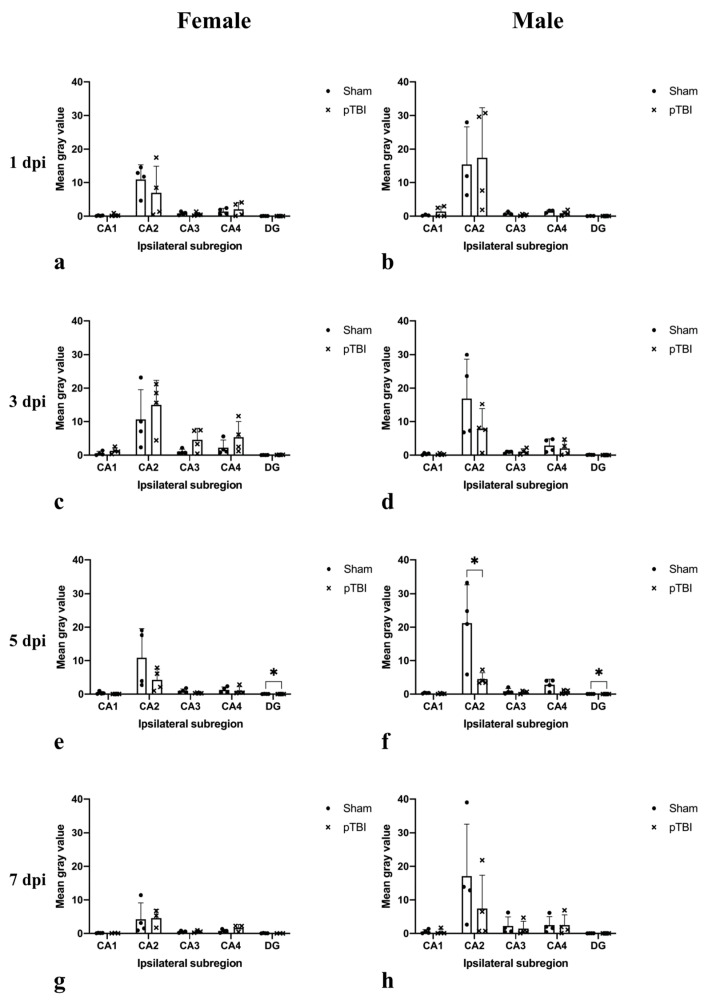
Comparison of hippocampal CYP1B1 mRNA expression detected by in situ hybridization in rats subjected to either sham-surgery or pTBI. Animals in Sham received a craniotomy, while animals in pTBI received a craniotomy with a subsequent pTBI. CYP1B1 mRNA was detected by in situ hybridization. Comparisons between Sham and pTBI were performed in animals with the same sex and survival time. Graphs show female mRNA expression 1 dpi (**a**), male mRNA expression 1 dpi (**b**), female mRNA expression 3 dpi (**c**), male mRNA expression 3 dpi (**d**), female mRNA expression 5 dpi (**e**), male mRNA expression 5 dpi (**f**), female mRNA expression 7 dpi (**g**), male mRNA expression 7 dpi (**h**). The mean gray value is presented in the Y-axis, showing the average mean gray value calculated from each measured cell for each subregion. To calculate mean gray value, images were made binary and in-situ probes made black and separated from the white background. The gray value of the color black was set to 255 and the value for white to 0. The gray value of each cell is therefore a measurement of the area of cell marked by the in-situ probe. The gray values from each cell of each subregion were then used to calculate the mean gray value of each subregion and animal. The X-axis shows the ipsilateral subregions analyzed. Quantified CYP1B1 mRNA was highest in subregion CA2 irrespective of sex, intervention, and survival time (**a**–**h**). 5 dpi, males subjected to pTBI exhibited significantly lower expression compared to sham in subregions CA2 (*p* = 0.0349) and DG (*p* = 0.0222) (**f**). In females 5 dpi, expression in DG was significantly lower in those subjected to pTBI compared to sham-surgery (*p* = 0.0264) (**e**). The values are presented as mean + SD for each group, including the mean for each animal. Horizontal bars marked with “*” show differences where *p <* 0.05. Abbreviations: CA: Cornu Ammonis, CYP1B1: Cytochrome P450 1B1, DG: Dentate gyrus, dpi: days post-injury, pTBI: penetrating traumatic brain injury.

In response to pTBI, all subregions exhibited altered CYP1B1 mRNA expression at some point. Both males and females subjected to pTBI exhibited an increased CYP1B1 mRNA expression in CA1 1 day post-injury (dpi) (Figure 2a,b). Unlike males, females subjected to pTBI had reduced expression in CA2 1 dpi compared to sham (Figure 2a,b). Females subjected to pTBI exhibited increased expression in all subregions compared to sham 3 dpi (Figure 2c). In contrast, males subjected to pTBI exhibited reduced expression in CA2 and CA4 3 dpi (Figure 2d). The increased expression observed in females with pTBI 3 dpi was reversed 5 dpi, and expression in CA1, CA2 and CA3 was lower than in sham counterparts (Figure 2e). 5 dpi, expression was significantly lower in males subjected to pTBI compared to sham-surgery in both CA2 (*p* = 0.0349) and DG (*p* = 0.0222) (Figure 2f). In females 5 dpi, significant reduction in the group receiving pTBI was only observed in DG (*p* = 0.0264) (Figure 2e). In males 7 dpi, expression in CA2 and CA3 was reduced in those subjected to pTBI compared to sham (Figure 2h). In females 7 dpi, no such decrease was observed and expression in those subjected to pTBI was increased in CA4 compared to sham (Figure 2g).

**Figure 3:**
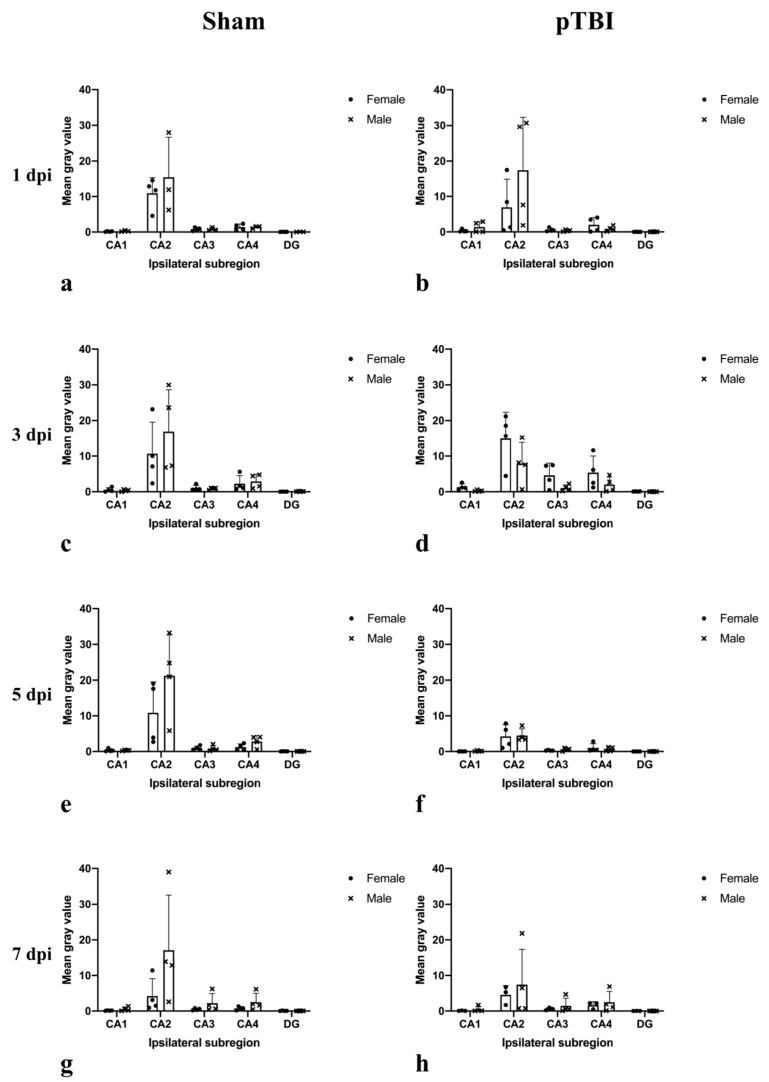
Comparison of male and female hippocampal CYP1B1 mRNA expression in rats subjected to either sham-surgery or pTBI. Animals in Sham received a craniotomy, while animals in pTBI received a craniotomy with a subsequent pTBI. Graphs show mRNA expression 1 dpi in males and females subjected to sham-surgery (**a**), mRNA expression 1 dpi in males and females subjected to pTBI (**b**), mRNA expression 3 dpi in males and females subjected to sham-surgery (**c**), mRNA expression 3 dpi in males and females subjected to pTBI (**d**), mRNA expression 5 dpi in males and females subjected to sham-surgery (**e**), mRNA expression 5 dpi in males and females subjected to pTBI (**f**), mRNA expression 7 dpi in males and females subjected to sham-surgery (**g**), mRNA expression 7 dpi in males and females subjected to pTBI (**h**). CYP1B1 mRNA was detected by in situ hybridization. The mean gray value is presented in the Y-axis, showing the average mean gray area calculated from each measured cell for each subregion. To calculate mean gray value, images were made binary and in-situ probes made black and separated from the white background. The gray value of the color black was set to 255 and the value for white to 0. The gray value of each cell is therefore a measurement of the area of cell marked by the in-situ probe. The gray values from each cell of each subregion were then used to calculate the mean gray value of each subregion and animal. The X-axis shows the ipsilateral subregions analyzed. Females subjected to pTBI and a 3 day survival time exhibited higher CYP1B1 mRNA expression in every CA-subregion compared to their male counterparts (**d**). The values are presented as mean + SD for each group, including the mean for each animal. Abbreviations: CA: Cornu Ammonis, CYP1B1: Cytochrome P450 1B1, DG: Dentate gyrus, dpi: days post-injury, pTBI: penetrating traumatic brain injury.

Sex-dependent differences in CYP1B1 mRNA expression were observed in rats subjected to sham-surgery. Expression in CA2 was higher in males compared to females at every time point studied (Figure 3). Similarly, expression in CA4 was higher in males 3, 5 and 7 dpi (Figure 3). Sex-dependent differences were also present in rats subjected to pTBI, where males exhibited higher expression in CA1 and CA2 compared to females 1 dpi (Figure 3b). In contrast, expression in females was higher in every subregion when compared to males 3 dpi (Figure 3d). The opposite was true when comparing expression 7 dpi, where expression in males was higher in every subregion compared to females (Figure 3h).

**Figure 4:**
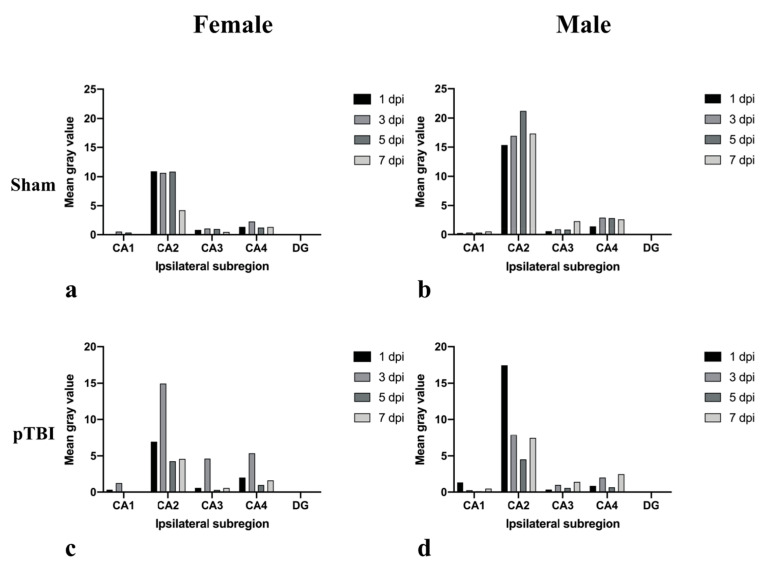
Overview of temporal changes in male and female hippocampal CYP1B1 mRNA expression in rats subjected to either sham-surgery or pTBI. Animals in Sham received a craniotomy, while animals in pTBI received a craniotomy with a subsequent pTBI. Survival times analyzed were 1-, 3-, 5- and 7 days. Graphs show timeline over CYP1B1 mRNA expression in females subjected to sham-surgery (**a**), males subjected to sham-surgery (**b**), females subjected to pTBI (**c**), males subjected to pTBI (**d**). CYP1B1 mRNA was detected by in situ hybridization. The mean gray value is presented in the Y-axis, showing the average mean gray area calculated from each measured cell for each subregion. To calculate mean gray value, images were made binary and in-situ probes made black and separated from the white background. The gray value of the color black was set to 255 and the value for white to 0. The gray value of each cell is therefore a measurement of the area of cell marked by the in-situ probe. The gray values from each cell of each subregion were then used to calculate the mean gray value of each subregion. The X-axis shows the ipsilateral subregions analyzed. In animals receiving pTBI, CYP1B1 mRNA expression is highest 1 dpi in males (**d**), whilst the highest expression in females is reached 3 dpi (**c**). The values are presented as means. Individual values and SD-values are presented in Figure 2, Figure 3 and Figure 5. Abbreviations: CA: Cornu Ammonis, CYP1B1: Cytochrome P450 1B1, DG: Dentate gyrus, dpi: days post-injury, pTBI: penetrating traumatic brain injury.

We observed differences in the temporal pattern of CYP1B1 mRNA expression. CA2 expression was lower in the females subjected to sham-surgery 7 dpi compared to 1, 3 and 5 dpi. Furthermore, an increase of expression occurred in CA4 3 dpi (Figure 4a). Temporal differences in expression among males subjected to sham-surgery were apparent. In CA2 and CA3 expression gradually increased and reached peak-expression after 5 and 7 dpi, respectively (Figure 4b). In CA4, lower expression was observed 1 dpi compared to other survival times (Figure 4b).

Temporal changes were greater in rats subjected to pTBI. In females, every subregion exhibited the same pattern of temporal changes, however the quantified levels differed. CYP1B1 mRNA expression was low 1 dpi but increased and reached peak-expression 3 dpi (Figure 4c). No apparent pattern of expression was observed in males, where CA1 and CA2 reached peak expression 1 dpi, followed by a sharp decline of expression 3 dpi (Figure 4d). Peak expressions in CA3 and CA4 were instead reached 7 dpi (Figure 4d). Similar to the temporal pattern of expression observed in females, every subregion examined in males had a higher expression 7 dpi than 5 dpi (Figure 4d).

**Figure 5:**
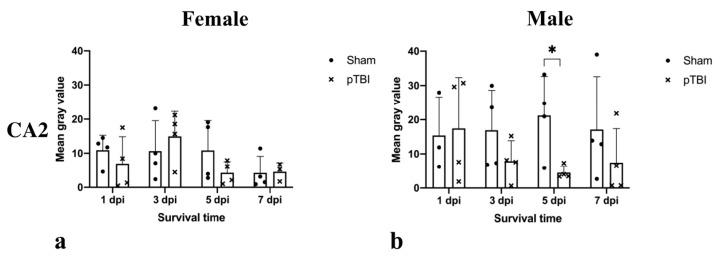
Comparison of CYP1B1 mRNA expression in hippocampal subregion CA2 in rats subjected to either sham-surgery or pTBI. Animals in Sham received a craniotomy, while animals in pTBI received a craniotomy with a subsequent pTBI. Survival times analyzed were 1-, 3-, 5- and 7 days. CYP1B1 mRNA was detected by in situ hybridization. Graphs show a timeline over CYP1B1 mRNA expression in subregion CA2 of females (**a**) and males (**b**) subjected to either sham-surgery or pTBI. The mean gray value is presented in the Y-axis, showing the average mean gray area calculated from each measured cell for each subregion and animal. To calculate mean gray value, images were made binary and in-situ probes made black and separated from the white background. The gray value of the color black was set to 255 and the value for white to 0. The gray value of each cell is therefore a measurement of the area of cell marked by the in-situ probe. The gray values from each cell of each subregion were then used to calculate the mean gray value of each subregion and animal. The X-axis shows the survival time. Males show no change in CYP1B1 mRNA expression 1 dpi, but 3-, 5-, and 7 dpi expression is decreased in the groups receiving pTBI compared to those receiving sham-surgery (**b**). 5 dpi, males subjected to pTBI exhibited significantly lower expression compared to sham in CA2 (*p* = 0.0349). The values are presented as mean + SD for each group, including the mean for each animal. Horizontal bars marked with “*” show differences where *p* < 0.05. Abbreviations: CA2: Cornu Ammonis 2, CYP1B1: Cytochrome P450 1B1, DG: Dentate gyrus, dpi: days post-injury, pTBI: penetrating traumatic brain injury.

Focusing on the subregion with the highest CYP1B1 mRNA expression, CA2, the response to pTBI differs between the sexes. In females subjected to pTBI, expression was lower than in females receiving sham-surgery 1 dpi, whilst no similar difference was observed in males (Figure 5). At 3 dpi, females subjected to pTBI exhibited higher expression than those receiving sham-surgery, while the opposite was true for males (Figure 5). Both sexes exhibited lower expression in the group receiving pTBI compared to those receiving sham-surgery 5 dpi, but this difference was only significant in males (*p* = 0.0349) (Figure 5). Males subjected to pTBI still had a lower expression compared to those receiving sham-surgery 7 dpi, whilst this difference was non-existent in females (Figure 5).

### 2.2. Immunohistochemistry

**Figure 6:**
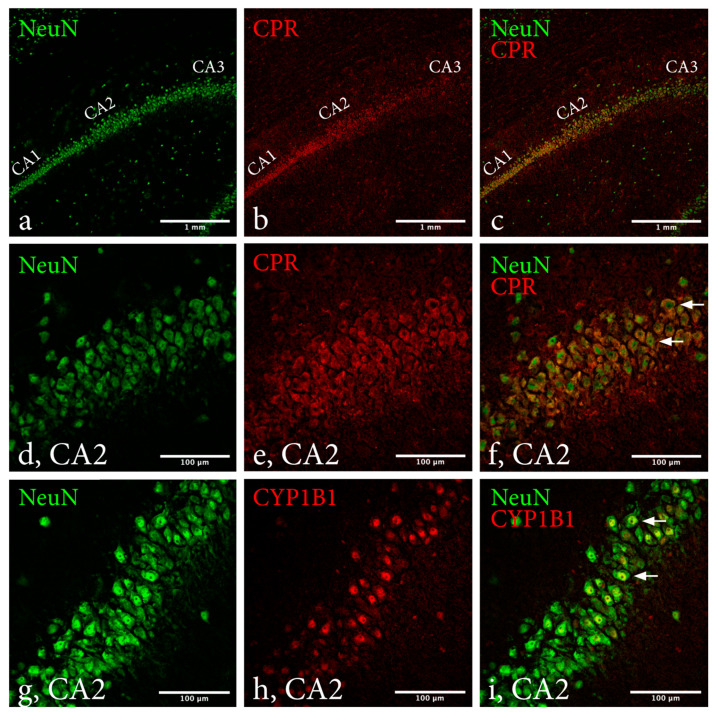
Immunohistochemical evaluation of CYP1B1 and CPR colocalization with NeuN in hippocampal subregions. Confocal images depicting an overview of CA1, CA2 and CA3 at 10× magnification (**a**–**c**), subregion CA2 captured at 40× magnification (**d**–**i**). Green channel (**a**,**d**,**g**) depicts NeuN staining, red channel (**b**,**e**,**h**) CPR or CYP1B1 and (**c**,**f**,**i**) the two channels merged. Distinct colocalization of CPR and NeuN was observed in subregions CA1 and CA2, but not CA3 (**c**). Positive colocalization of CYP1B1 and NeuN was noted in several cells, indicating presence of CYP1B1 protein in CA2 neurons (**i**). Examples of cells judged as positive for CPR or CYP1B1 are marked by arrows. The image was deemed representative of all groups, as no apparent difference was observed between sexes, interventions, or survival times. Abbreviations: CA2: Cornu Ammonis 2, CPR: Cytochrome P450 reductase, CYP1B1: Cytochrome P450 1B1.

Regardless of sex, intervention and survival time, qualitative analysis determined colocalization of CYP1B1 and NeuN in every hippocampal subregion. No apparent differences were noted between subregions or groups stated above (Figure 6). For CPR and NeuN, distinct colocalization was only observed in subregions CA1 and CA2. This was true regardless of sex, intervention, and survival time. Regarding colocalization in CA1 and CA2, no apparent differences were observed between the groups.

### 2.3. Estrus Cycle Staging

Estrus cycle staging was performed on all 31 females using standard hematoxylin-eosin staining. In females receiving sham-surgery, 7 were staged in phases of the estrus cycle characterized by low estradiol levels while 10 were staged as proestrus, a phase characterized by high E2 levels. In females subjected to pTBI, 5 were staged in phases characterized by low E2 levels while 9 were staged as proestrus, characterized by high E2 levels.

## 3. Discussion

CYP1B1 metabolizes the neuroprotective sex hormones 17β-estradiol (E2) and progesterone and modulates the inflammatory response by producing HETEs [12,30,31]. CYP1B1 activity directly correlates to its electron donor Cytochrome P450 reductase (CPR). Female rodents exhibit superior outcome following TBI and multiple sequala are linked to hippocampal dysfunction [32,33,34].

Therefore, this study aimed to describe hippocampal CYP1B1 mRNA expression as well as protein presence of CYP1B1 and CPR in rats subjected to pTBI and determine if there were indications of sex-dependent differences.

The study is underpowered due to the lack of previous information regarding CYP1B1 mRNA expression in relation to the hippocampal subregions and TBI. Subregional CYP1B1 mRNA expression has not been previously studied and we could therefore not perform a reliable power analysis, and instead performed an experimental pilot study aiming at identifying key timepoints for future studies. Post-hoc analysis based on the results presented in this article show close to a total 200 animals would be necessary assuming the same number of groups, alpha = 0.5 and 80% power. We therefore intend to perform future studies with increased power, aimed at targeted timepoints to limit the number of animals used.

In this study, our results show homogenous presence of CYP1B1 protein while CPR is predominantly concentrated to subregions CA1 and CA2. Furthermore, we show CYP1B1 mRNA expression in every hippocampal subregion before and after pTBI, with distinct interregional differences and possible sex-dependent differences in expression.

CYP1B1 mRNA expression in every hippocampal subregion irrespective of intervention was expected, as E2 and progesterone are vital for brain function [35,36]. For every group, expression was greatest in CA2 and lowest in DG. These findings were confirmed using ViewRNA as an alternate method for mRNA in situ-hybridization. Interestingly, the distinct presence of CPR in subregions CA1 and CA2 are in line with our findings of CYP1B1 mRNA possessing interregional differences in expression with a concentration to CA2. As a key redox partner to CYP1B1 necessary for its activity, the concentration of CPR to subregion CA2 further implies importance of CYP1B1 in CA2 compared to other subregions.

Males subjected to sham-surgery exhibited a higher expression in CA2 at every timepoint when compared to females, possibly explained by sex-dependent differences in hippocampal E2 concentrations. In contrast to peripheral E2, hippocampal E2 concentrations are higher in males than in females [37]. Additionally, previous research has shown CA2 exhibits differences in genetic expression profile compared to other subregions [26], most notably genes linked to growth factor signaling and signal transduction.

Our results indicate that changes in CA2 CYP1B1 expression in pTBI are sex dependent. Expression 1 dpi was unaffected in males whilst reduced in females. Male peak expression occurred 1 dpi, followed by reduced expression 3, 5 and 7 dpi. Females reached peak expression 3 dpi and no difference in expression was noted 7 dpi. Previous studies have described sex-dependent divergence in acute neuroinflammatory response to TBI, males having a more acute inflammatory response than females [38,39]. CYP1B1 mRNA is induced by cytokines like TNF-alpha and aryl hydrocarbon receptor ligands [40], this inflammatory divergence could contribute to differences observed in CYP1B1 expression. However, our results suggest that the overall effect of pTBI on CYP1B1 is reduced expression, indicating that other regulatory factors counterbalance induction driven by neuroinflammation.

Males subjected to sham-surgery exhibited higher CYP1B1 mRNA expression in CA2 at every timepoint compared to females. However, after pTBI female expression was higher 3 dpi and equal to male expression 5 dpi. Extracerebral CYP1B1 is regulated by both hormonal factors and through the aryl hydrocarbon receptor [41]. Both estrogens and progesterone can induce CYP1B1 mRNA expression and protein synthesis in fibroblasts [42]. If applicable to hippocampal neurons, increase of female CYP1B1 mRNA expression in CA2 may be due to increased BBB-leakage occurring following TBI [43], allowing estrogen and progesterone to enter the CNS. CYP1B1 is involved in BBB-permeability, and the altered expression following injury may influence BBB integrity [44]. Our model causes increased BBB-leakage, peaking 3 dpi and thereafter declining [28]. As peripheral E2 and progesterone concentrations are higher in females than males, it could possibly affect females more than males. In this study, females lacked synchronized estrus cycle, resulting in too small populations to draw statistical conclusions regarding correlation of CYP1B1 mRNA expression and estrus cycle phase.

The penetration model used lacerates the fornix, depriving the hippocampus of input, resulting in impaired spatial memory function [45]. The laceration occurs rostrally of the fimbria projecting to the hippocampal subregions, affecting afferent input and retrograde atrophy in all subregions to equal extent. Nonetheless, effects of this altered input may differ regarding CYP1B1 expression, as CA2 possesses an innate resistance to ischemia and TBI relative to other subregions [26].

The effect of altered CYP1B1 expression is beyond the scope of this study. There is however evidence of both positive and negative effects of altered CYP1B1 protein levels indicating that CYP1B1 acts on both beneficial and deleterious pathways. A previous study [5] showed that CYP1B1 deficiency resulted in increased oxidative stress and reduced angiogenesis. On the contrary, there are evidence of 20-HETE-antagonists reducing lesion area in ischemic injuries [14]. Furthermore, CYP1B1 metabolizes E2, which besides its neuroprotective properties [40,41,42,43] is essential for hippocampal functions frequently effected by TBI [35]. CYP1B1 also metabolizes progesterone [4], which possesses similar neuroprotective and anti-inflammatory properties as E2 but is also involved in the pathophysiology of posttraumatic epilepsy [46]. Treatment of posttraumatic epilepsy may be altered depending on CYP1B1 protein levels as susceptibility to anti-epileptic drugs alters with CYP1B1 single-nucleotide polymorphism [13]. Moreover, CYP1B1 catalyzes both steps in the production of retinoic acid from retinol [4]. Retinoic acid possesses several neuroprotective traits after TBI [47] and contributes to hippocampal neurogenesis [48]. In this study we suggest CYP1B1 mRNA is expressed in all hippocampal subregions with distinct interregional differences. However, future studies with increased power will be required to confirm our preliminary findings, and thereafter if altered CYP1B1 expression influences inflammatory and neuroprotective molecules in pTBI.

## 4. Materials and Methods

The study was approved by the Swedish regional ethics approval board for animal research (N244/15).

### 4.1. Material

A total 32 male and 32 female adult Sprague Dawley rats received sham-surgery or pTBI and were designated a survival time of 24-, 72-, 120- or 168 h. Each group contained four male and four female rats (Table 1). One male rat exposed to sham-surgery and a survival time of 24-h was excluded due to brain malformation and one female rat subjected to pTBI and a survival time of 168-h was excluded due to contralateral cortical injuries observed during organ harvesting. Males weighed between 408 and 512 g (mean = 451.21 g; SD = 22.11 g) and females weighed between 276 and 365 g (mean = 323.09 g; SD = 17.56 g). Rats were kept in standard sized cages divided by sex in groups of two. A twelve-hour light/dark cycle was employed, and animals had free access to water and pellets.

### 4.2. Statistics

As the nature of the study is explorative and a pilot for future studies, group sizes were small and underpowered to reduce the number of animals used.

Statistical analysis was performed using GraphPad Prism 9 (GraphPad Software, San Diego, CA, USA). A two-way ANOVA followed by a Tukey multiple comparisons test was performed to analyze the effect of sex and intervention on CYP1B1 mRNA expression. Alpha level was set to 0.05 and *p*-values < 0.05 were considered significant.

Post-hoc power analysis showed the study was underpowered and means were calculated for sham-surgery compared to pTBI (mean = 0.296, SD = 0.19), sex-differences following sham-surgery (mean = 0.16, SD = 0.13) and pTBI (mean = 0.26, SD = 0.19).

### 4.3. Surgery and Penetrating Trauma

**Figure 7:**
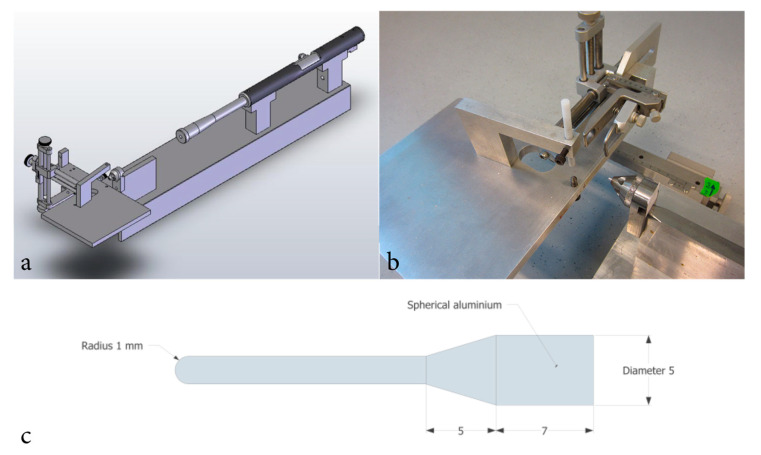
Schematic illustration of the equipment used for inducing penetrating traumatic brain injury. Schematic illustration of the trauma model (**a**), image of the stereotactic frame, animal platform and cone formed probe guider (**b**). Schematic illustration of secondary impactor probe design (**c**).

Animals were anaesthetized in a sealed chamber containing 4% isoflurane. Thereafter, animals were equipped with a nose mask continuously supplying 2.0 mL/h isoflurane and placed on a heated surgical platform. After midline incision through the skin and periosteum, a drill was used to create a 2.75 mm bore hole 2 mm posterior and 2 mm lateral of bregma, exposing the dura. Animals were then placed in a stereotactic frame with a secondary projectile adjacent to the dura. The secondary projectile consisted of a 30 mm long probe constructed in one single unit in aluminum. The penetrating pins diameter was 2 mm and its tip spherical. The probe weighed 0.66 g [27]. A modified air-rifle (CNC-Process AB, Hova, Sweden) was used to fire a lead pellet that impacted the secondary projectile which accelerated to a speed of 110 m/s as it lacerated meninges and brain parenchyma. A cone shaped section of the secondary probe limited mean penetration depth to 5.12 mm (n = 32; SD = 0.06 mm). For animals subjected to sham-surgery, a blank bullet was used and thus no penetration occurred. Lastly, the skin was sutured, and the animals were returned to their cages. A schematic illustration of the model is presented in Figure 7. Previous studies describing the penetration model in further depth are available [27,28].

### 4.4. Tissue Sectioning

After sacrifice, brains were removed and fresh frozen on dry ice until placed in a −70 °C freezer. Brains were cut into 14 µm coronal sections using a Thermo Fisher NX70 cryostat and mounted onto Thermo Fisher Superfrost plus glass and stored in −70 °C. Sections used were located between Bregma: −3.84 mm and Bregma: −4.80 mm.

Removal of female reproductive organs was performed in conjunction with animal sacrifice and the tissue was placed in formaldehyde and thereafter a 5 °C refrigerator overnight. 14 µm sections were then cut in a Thermo Fisher NX70 cryostat and mounted onto Thermo Fisher Superfrost plus glass.

### 4.5. In-Situ Hybridization

mRNA in-situ hybridization was performed using both RNAscope (ACD, Santa Ana, CA, USA) and ViewRNA (ThermoFisher Scientific, Carlsbad, CA, USA), for both methods the companies recommended probe aimed at targeting CYP1B1 mRNA was used.

The RNAscope technique utilizes paired oligonucleotide target probes with a double-Z design and subsequent signal amplification to visualize single mRNA molecules [49]. In-situ hybridization was performed according to the manufacturer’s instructions with the sole exception being a reduced proteas time from 30 min to 25 min. Target probe used was Rn-CYP1B1 (ACD, Catalog No: 493211) and reagent RNAscope^®^ 2.5 HD Detection Reagents-BROWN (ACD, Catalog No: 322310). After Cresyl violet counterstaining mounting was performed with Entellan. 

ViewRNA is an alternative method for mRNA in-situ hybridization and was used as a positive control to RNAscope. In contrast to RNAscope, ViewRNA utilizes oligonucleotides combined with branched DNA for signal detection and amplification [50]. ViewRNA Tissue Assay Core Kit (ThermoFisher Scientific, Catalog No: 19931) and target probe Rat CYP1B1 (ThermoFisher Scientific, Catalog No: VPU62U6) were used according to the manufacturer’s instructions and counterstained using methyl green.

### 4.6. Estrus Cycle Determination

Tissue from the ovaries, uterus and vagina were stained with hematoxylin-eosin according to standard protocol. The estrous cycle stage was determined by applying the criteria described by Westwood [51].

### 4.7. Immunohistochemistry

Immunohistochemistry was performed on 14 µm coronal brain sections. Slides were placed in room temperature for 30 min, and thereafter placed in 4% buffered formaldehyde for 10 min. Slides were then rinsed in 0.01 M PBS and incubated in a humid chamber at 5 °C for 21 h with either CYP1B1 antibody (Abcam, Product ID: ab185954) diluted 1:2000 or CPR antibody (Abcam, Product ID: ab180597) diluted 1:100 together with NeuN antibody (Millipore, Catalog No: MAB377) diluted 1:1000, 5% donkey serum and 95% primary buffer (0.01 M PBS + 0.1% NaN3 + 0.3% triton + 5% bovine serum albumin). Thereafter, slides were rinsed in 0.01 M PBS and incubated in room temperature for 30 min with secondary antibodies Cy3 (Jackson ImmunoResearch, Product ID: 711165152) and Alexa Flour 488 (Jackson ImmunoResearch, Product ID: 715545150), both diluted 1:400 with secondary buffer (0.01 M PBS + 0.1% NaN3 + 0.3% triton). Thereafter slides were rinsed in 0.01 M PBS and mounted with Mowiol.

### 4.8. Image Analysis

**Figure 8:**
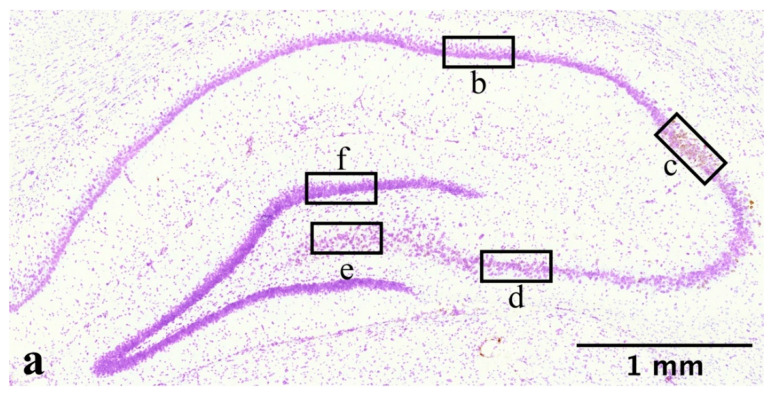
Overview of hippocampal anatomy and locations of image acquisition. Stitched image (**a**) of ipsilateral hippocampus of female rat subjected to penetrating traumatic brain injury and a survival time of 3 days. Acquired at 20× magnification using a Nikon Eclipse Ni-E microscope. Acquired at Bregma: −3.84 mm. RNAscope in-situ hybridization marked CYP1B1 mRNA in brown. Black boxes (**b**–**f**) show location and scope of image captured as well as CYP1B1 mRNA markings. Box (**b**) depicts CA1, (**c**) CA2, (**d**) CA3, (**e**) CA4 and (**f**) DG. Abbreviations: CA: Cornu Ammonis, CYP1B1: Cytochrome P450 1B1, DG: Dentate Gyrus.

Images used to analyze the in-situ hybridization were acquired at 20× magnification using a brightfield Nikon Eclipse Ni-E microscope equipped with a Nikon-Fi2 camera. Imaging locations were pre-decided and are described in Figure 8. Images were analyzed in ImageJ/Fiji. Each granule and pyramidal cell were marked with a region of interest (ROI) corresponding to the average cell size of the subregion (CA1 = 1880 pixels; CA2 = 2162 pixels; CA3 = 2644 pixels; CA4 = 2372 pixels and DG = 1456 pixels). Images were then made binary, and a manual threshold was applied by a blinded assessor. Thereafter, the mean gray value for each marked ROI was used to calculate the subregions average mean gray value. Data was transferred to GraphPad Prism 8.

To assess CYP1B1 and CPR colocalization with NeuN, a Nikon Eclipse E600 equipped with a Nikon C1 confocal system with an Argon Ion Laser 488 nm (40 mW) and a HeNe Laser 543 nm (1.0 mW) was used to acquire images at 40× magnification. Qualitative analysis of the images was performed by a blinded assessor.

## 5. Conclusions

CYP1B1 mRNA is expressed in every hippocampal subregion with indications of sex-dependent and interregional differences. Furthermore, we show these differences may persist after pTBI. CYP1B1 expression was greatest in CA2 regardless of sex and intervention. We also suggest that the temporal pattern of expression following pTBI exhibits indications of sex-dependent differences regarding time of response, peak expression, and normalization. Immunohistochemical analysis showed CYP1B1 protein in neurons of all hippocampal subregions irrespective of sex, while CPR colocalization was concentrated to CA1 and CA2. If our results are confirmed by future studies and altered CYP1B1 mRNA expression correlates to levels of CYP1B1 protein, male and female rodents may have a different inherent neuroprotective and inflammatory response to pTBI. Furthermore, the concentration of CYP1B1 mRNA and CPR protein to CA2 may indicate an unknown importance of CYP1B1 activity for CA2 function.

## Figures and Tables

**Table 1:** Material composition based on sex, survival time and intervention.

Intervention	Survival Time	Number of ISH	Number of IHC
Sham-surgery	24 h	Male = 3, Female = 4	Male = 3, Female = 4
pTBI	24 h	Male = 4, Female = 4	Male = 4, Female = 4
Sham-surgery	72 h	Male = 4, Female = 4	Male = 4, Female = 4
pTBI	72 h	Male = 4, Female = 4	Male = 4, Female = 4
Sham-surgery	120 h	Male = 4, Female = 4	Male = 4, Female = 4
pTBI	120 h	Male = 4, Female = 4	Male = 4, Female = 4
Sham-surgery	168 h	Male = 4, Female = 4	Male = 4, Female = 4
pTBI	168 h	Male = 4, Female = 3	Male = 4, Female = 3

Abbreviations: ISH: In situ hybridization, IHC: Immunohistochemistry, pTBI: Penetrating traumatic brain injury.

## Data Availability

Data is available through Mendeley Data, at doi:10.17632/dpp455r2jv.1.

## Abbreviations

AA	arachidonic acid
BBB	blood-brain-barrier
CA	Cornu Ammonis
CPR	Cytochrome P450 reductase
CYP	Cytochrome P450
CYP1B1	Cytochrome P450 1B1
DG	Dentate Gyrus
dpi	days post-injury
E2	17β-estradiol
HETEs	hydroxyeicosatetraenoic acids
IHC	immunohistochemistry
ISH	in situ hybridization
pTBI	penetrating traumatic brain injury
ROI	region of interest
TBI	traumatic brain injury

## Appendix A

**Table A1:** Data extracted from gen array of whole hippocampi and cortical tissue from rats subjected to sham-surgery or pTBI [3].

	CYP1A1	CYP1A2	CYP1B1	CYP2A1	CYP2A2	CYP2E1	CPR
Hippocampus pTBI vs. Sham Fold change	1.30	1.16	15.03	1.11	1.10	1.17	1.99
Cortex pTBI vs. Sham Fold change	1.08	1.04	4.65	1.08	−1.18	1.08	−1.63

Abbreviations: CYP1A1: Cytochrome P450 1A1, CYP1A2: Cytochrome P450 1A2, CYP1B1: Cytochrome P450 1B1, CYP2A1: Cytochrome P450 2A1, CYP2A2: Cytochrome P450 2E1, CPR: Cytochrome P450 reductase, pTBI: Penetrating traumatic brain injury.
